# Malignant Disease of the Skin

**Published:** 1916-05

**Authors:** W. L. Crosthwait

**Affiliations:** Waco, Texas


### Malignant Disease of the Skin.

DR. W. L. CROSTHWAIT, WACO, TEXAS.

When we come to discuss any phase of malignancy nowadays we are reminded of the old saying, "It is not what we don't know, but what we do know that is not so, that makes us ridiculous." These few things about cancer we are positive:

First. It has an acute and definite beginning.

Second. It is at first a local disease.

Third. It metastasis, beginning at an indeterminate time after its implantation or incipiency.

Fourth. It can be cured if treated properly at the right time.

Fifth. It is always preceded by a characteristic abnormality or lesion.

Sixth. It can be prevented by removing or curing the lesion that favors implantation.

Beyond these few elementary facts we have not progressed. Beyond these few facts nothing positive has been proven. Theories galore have been advanced, volumes have been written based upon inadequate experimentations, erroneous observations and doubtful deductive reasoning, but no scientist has expressed more than a belief as to its etiology based upon facts, experiments or observations worthy of acceptance. Therefore it will not be amiss if we discuss the subject of malignancy of the skin in the light of the few facts which are proven or generally accepted as proven, and, furthermore, in the theoretical way in which we will attempt to discuss some of the things which we do not know and some of the things we do know which may or may not be so.

Malignant disease of the skin may appear on any part of the cutaneous surface, although it is rare on the limbs, hands or feet, having certain parts of the body as predilective or selective locations, mostly near the site of the muco-cutaneous margins, or at some point where chronic lesions or ulcers are subjected to long and continued irritations, traumatism and infections.

Histologically speaking, skin cancers may arise from any layer of the surface or epithelium. It may also come upon any structure in the skin from the hair follicles and sebaceous and sweat glands, etc. The new growth takes on a varied cellular growth, the most important of which is the prickle-cells, the basal-cells, and the nevocarcinoma.

This is purely a technical classification and proves nothing as to the etiology of the disease. It merely indicates a particular characteristic of malignant growth to partake of the histological structure of the particular cells which first become infected. In the prickle-cells type it is reasonable to presume that these cells would become easily inoculated or impregnated with numerous germs or virus for the reason that they are crippled to a certain extent as they become keratosed and lose greatly in vitality in the process of forcing themselves from the middle epithelium layer towards the surface. The basal-cells are easily susceptible of invasion for the same reason. Cuboidal-cells predominate around the margin of ulcers and other lesions which are the precursors. The nevocarcinoma or malignant wart is probably malignant from the beginning, passing through an indefinite period before the cells have the inherent power to intrude themselves into contiguous tissue, or to take on a characteristic or malignant type. A comparative study of the malignant wart from its first appearance until it become clinically or definitely pathologically malignant, would be both interesting and instructive. If such study has been made, the author has been unable to find it in current literature. We will, therefore, pass to a clinical description of some of the lesions of the skin, which afford most favorable grounds for malignation of cancerous growth.

The prickle and basal-celled carcinoma both seem to have a predilection to implant themselves upon the site of a lesion of a more or less inflammatory nature, such as an ulcer, burn, inflamed wart, Roentgen-Ray, dermatitis or arsenical or sinite keratosis, indolent pimple, or around the margin of a sinus.

The cuboidal-celled carcinoma may grow from any of these conditions, but seems to most often select the margin of an ulcer, chronic sore or burn, a senile keratosis which is subjected to irritation and from location or position is kept moist from the body secretions, such as lesions on the hips of old persons and bed sores, thus becoming infected quite frequently, no doubt, from soiling with feces.

The carcinoma coming from the so-called malignant warts, the writer has expressed an opinion that this type is malignant from the beginning, the cancer germs being directly implanted in the tissue from some insignificant or unnoticed cause, and like tetanus bacilli, remains an indefinite time before beginning to show growth in the form of a wart or mole and a certain other indefinite time before showing characteristic carcinomatous growth.

It has been shown that cancer infection takes place around the margin of an ulcer or lesion. The reason for that may be due to one or two causes. First, the chemolysis resulting from cell destruction may produce a toxine noxious or destructive to the cancer germ. Second, the peculiar habit of certain cells to move away from antagonistic agents and arrange themselves around the outside border of the danger zone. This last reason is most probable because in such lesions as usually precede cancer we find a certain type of epithelial cells abundant around the margin of such lesions as are most often characteristic of carcinomatous growth, and we further find a break in the tissue at that point making a favorable egress for the cancer germs.

The next question is what conditions are most favorable to the development of carcinoma.

First. Age of individual. It is now claimed that age has no specific bearing on the development of cancer. Carcinoma is known to develop in individuals of all ages.

Second. Type of individual. This factor plays quite an important role, quite analogous to other specific infections. We would deprecate the use of the term pre-cancerous condition as descriptive of individuals thought to be most susceptible of becoming cancerous. There must be potential causes aside from hereditary influences to account for the fact that one individual will carry a certain lesion for an indefinite time as benign and another individual will carry the same kind of lesion under identical conditions of irritation, trauma and opportunity for infection developing malignancy.

Third. Mode of life, occupation, etc. There is no doubt but what the person whose occupation compels him to handle dirt, manure and other filth is the more liable to cancer. Cats, poodle dogs, parrots, pet rats and other animals that eat decayed carnivorous food are cancer carriers and often, no doubt, infect their wealthy owners. Persons who eat poorly cooked and badly selected meat, raw vegetables from barnyard gardens often get cancer implanted upon their lesions or other so-called pre-cancerous lesions.

Fourth. Location of lesion. The location of the so-called precancerous condition has undoubtedly much to do with the implantation or development of cancer. This, though, carries with it special reference to continued irritation or traumatism, but with more especial consideration as to its exposure to repeated or protracted chances to become infected.

It is obvious from what we have said that we believe that all malignancy is of an infectious nature. The persistent pipe smoker first gets an ulcer, then a hyperplasia of epithelium, which becomes ulcerated and infected from the secretions of the mouth. The bottle nut chewer first has an ulcer which becomes infected from the saliva of the mouth. The woman with Paget's nipple has first an ulcer, which may be infected by the saliva of a nursing child, after it has begun to take other food, or from other sources. The person with an ulcer on his face carries his open sore or ulcer until it may be infected from the hands, or from a thousand other sources.

The last but most important proposition is the early recognition of the lesions likely to become the site of carcinomatous development and their complete removal or cure. The suspicious or suspected growth or condition should be freely excised and a specimen submitted to a competent pathologist, and if found to be malignant the neighboring glands should be removed for fear metastasis has begun. The fact is too well established that malignancy in its early stages is amenable to surgical cure and the technic is well understood by both surgeons and practitioners, that a discussion of that phase of the subject is hardly worth while before this society. We will, therefore, pass to the theoretical part of our subject.

For many years the leading surgeons and pathologists have thought that carcinoma was due to a continued or chronic irritation and that sarcoma was due to a single irritation. These are at most only theories, the outcome of deductive reasoning. The test of a theory is its practical application and the absolute proof of a deductive conclusion is its practical demonstration when applied to concrete facts.

No experimenter has been able to produce carcinoma by the application of irritation alone, as far as I know. In other words, no experimenter has been able to produce a carcinoma by any kind of irritation, external or internal, under conditions where all other possible etiological factors have been excluded. No one has been able to produce a sarcoma by the application of a single irritation under conditions where all other etiological factors have been eliminated. It is probably impossible for this to be done in either case, and for that reason our conclusions on these matters must remain theoretical and not proven.

The theory has been advanced that carcinoma is not due to a continued irritation of a single or particular kind of nature, but that it is produced by a defensive reaction of the cells to a variety of irritation, whether such irritations be microbic, traumatic, thermic, toxic or radiant. This theory is less demonstrable than the first and in reality has for its foundation the embryonal cell theory. Of course it is true that when an irritation of any kind falls short of death of the cells in its effect, there follows a defensive cell proliferation. An active fight is on between the destructive agency and the Karo Kinetic power of the cells. We may admit that the more evenly balanced this contest may be, the more near the cells approach the embryonal in type. But it does not necessarily follow that in this approximately evenly balanced contest that new cells spring up from the epiblast or from below the basement membrane and engraft themselves upon those from the mesoblast, which cells have become crippled or impaired, and thus by such union become cancer cells with far greater inherent power than either parent cells have possessed formerly.

In other words, it is a stretch of the imagination quite beyond the writer to conclude that a cell or multiplicity of cells could in the process of reversion or evolution which if present is due or is the result of a destructive process, could ever become so powerful as to defy every principle of development, growth, life, defensive and offensive faculties of normal cells.

In the etiology of cancer nothing has been proven.

If left to the vagueness and uncertainty of deductive reasoning we would prefer to account for the origin of carcinoma from infective or microbic sources. Drs. Wilson and McCarty have shown that in a large percentage of carcinoma following ulcer of the stomach, that not the base of the ulcer, but overhanging margins become carcinomatous. Other pathologists have shown that carcinomatous cells could be positively identified only along the margin of ulcers.

In carcinoma of the cervix following long standing ulceration or lacerations, ebrasions, etc., show carcinoma cells along the border or margin of the old lesion. It has also been shown that carcinoma of the face that becomes engrafted upon the side of ulcers or erosion, that carcinoma cells could only be found along the margin of the old lesion where sections have been made in the early stages of carcinomatous growth.

The failure of investigators acting independently and jointly, individually and as members of the various cancer research organizations, etc., to prove the approximate correctness of any of the proposed theories concerning the essential causes of malignant disease, has resulted in the concentration of thought upon factors or conditions, which might be considered or which might be proven as predisposing factors as concerns the etiology of cancer.

The writer does not concede that there has been or will be a characteristic or standardized chain of symptoms which could correctly be termed pre-cancerous symptoms, but that there may be a standardized and fairly pathognomonic chain of symptoms evidencing certain lesions or conditions, which are most favorable to the implantation and development of malignant disease. As far as symptomatology is concerned, we would prefer not to use the term pre-cancerous symptoms, as descriptive of incipient cancer, rather choosing the term inaugural or early symptoms.

We speak here advisedly for the obvious reason that so great a variety of conditions are potential factors in the development of malignant disease. As, for instance, we note in the study of malignant disease of the skin, numerous types of chronic lesions, epithelial growths, indolent ulcers and persistent abrasions and chronic fissures, of which only a limited proportion become the site for implantation of cancerous growth. If we apply the term pre-cancerous condition to the lesion which ultimately develops into cancer, or which ultimately become the site for implantation of cancerous growth, we would find ourselves going to the far extreme of classifying every lesion of the body whether external or internal and every point of irritation as a pre-cancerous condition. It appeals to the writer as being more logical and in accord with the well established scientific methods of modern diagnosis to study and classify these lesions and conditions in reference to their etiology, duration, and location and classify them as predisposing factors with special reference as to the proven per cent of malignant development which obtains under fixed conditions.

Determining factors in this classification should include predilective influences, such as heredity, environment, mode of life and personal habits, type, duration, and location of lesion, and exposure to continued irritation, traumatism and infection. In other words, any benign growth or lesion of the body enters into the consideration of the etiological factors of cancer only in the light of their potentiality of affording suitable fields for the implantation of cancer germs.

The "border line" between benign and malignant is sharply drawn. It is not a wide zone like unto the territory between the tropics of Cancer and Capricorn, but like unto the equator dividing the continent into the North and South Hemispheres. To my mind it is as meaningless to say that a person was in a pre-typhoid state all the years he had been eating and drinking typhoid germs before contracting the disease, or to say that a woman was in a pre-pregnant condition all the days of the honeymoon or intervening month before she becomes pregnant, as to say that a man with an ulcer or sore or mole on his lip was in a pre-cancerous condition all the months or years prior to his infection or inoculation with cancerous germs. The doctors should teach, for the world should know and some day will know, that a patient's lesion is either benign or malignant, that he has cancer or does not have it; that cancer has a precise, definite and acute beginning.

The people should know that the removal of an abnormality will not cause cancer to develop, if such abnormalities or ulcer is not cancerous at the time of the operation. Patients frequently refuse to have an indolent mole or ulcer or other abnormalities treated surgically for fear that such treatment will cause cancer to develop. He should know that the way to prevent cancer is a complete and proper removal of such condition. What is meant by the complete and proper removal of such abnormality, is the removal of the entire growth, not a portion of it. Whenever it is thought advisable to operate on a condition for fear it might become malignant, it is certainly advisable to remove it entirely, immediately submitting a specimen for pathological examination, which, if found malignant, should be further treated by removal of the lympathic glands in the area, draining into region of growth.

